# Prosthetic valve endocarditis due to *Candida parapsilosis* – a rare case report

**DOI:** 10.1099/acmi.0.000462.v4

**Published:** 2023-01-24

**Authors:** Shivang Sharma, Subhashree Samantaray, Deepak Kumar, Durga Shankar Meena, Rahul Chaudhary, Vidhi Jain, Gopal Krishana Bohra, Mahendra Kumar Garg

**Affiliations:** ^1^​ Department of General Medicine (Infectious Diseases), All India Institute of Medical Sciences, Jodhpur, Rajasthan, India; ^2^​ Department of Cardiology, All India Institute of Medical Sciences, Jodhpur, Rajasthan, India; ^3^​ Department of Microbiology, All India Institute of Medical Sciences, Jodhpur, Rajasthan, India

**Keywords:** endocarditis, *Candida parapsilosis*, combined surgery and antifungal treatment

## Abstract

Fungal endocarditis is a rare and fatal condition**,** most frequently caused by species of the genera *Candida* and *Aspergillus*. Fever and changing heart murmur are the most common clinical manifestations. The diagnosis of fungal endocarditis is challenging, with prosthetic valve endocarditis being extremely difficult to diagnose. The optimal management of the condition still remains debatable. We present a case of prosthetic valve endocarditis caused by *Candida parapsilosis*, managed empirically with liposomal amphotericin B, which was later shifted to combination therapy with high-dose echinocandin and fluconazole, but had a fatal outcome because the patient could not undergo timely surgical intervention. Treating *C. parapsilosis* endocarditis cases is difficult because of their biofilm production on native and prosthetic heart valves. A combined approach consisting of a high index of clinical suspicion, early diagnosis using serological markers followed by culture or PCR and prompt initiation of appropriate antifungals may aid in improving outcomes.

## Data summary

No new data were generated in the study.

## Introduction

Infective endocarditis (IE) is challenging to diagnose, having many presentations, ranging from an indolent infection to septicemia with life-threatening systemic embolizations [1]. The incidence of fungal endocarditis (FE) has been increasing, accounting for 1.3–6 % of all reported IE and ~3 % of prosthetic valve endocarditis cases, with a mortality rate as high as 50 % [2, 3]. The two most common fungi causing endocarditis are species of the genera *Candida* and *Aspergillus*, usually isolated from surgically removed emboli, resected valves or infected foreign bodies [4]. Even though *Candida albicans* is the most common *Candida* species causing FE, the non-albicans *Candida* (NAC) species have also contributed to significant in-hospital mortality and morbidity [5]. Among the NAC species, incidence of *C. parapsilosis* IEhas increased in the past two decades [2, 6]. Such patients most commonly present with fever and changing or new onset cardiac murmurs [7]. The common risk factors associated with FE are underlying cardiac abnormalities, prosthetic heart valves, previous cardiac surgery, intravenous drug abuse and other immunocompromised conditions predisposing to invasive candidiasis [4]. The diagnosis of FE is often difficult because of its close resemblance to bacterial IE. Additionally, treating the condition after diagnosis is often difficult because of the ability of the *Candida* species to form biofilms on native and prosthetic valves, causing poor penetration of antifungal agents [8]. Hence a combination of source removal and prolonged antifungal therapy is recommended for the treatment of FE cases. We hereby report a case of prosthetic valve endocarditis caused by *C. parapsilosis* and managed with a combination of high-dose caspofungin and fluconazole that had a fatal outcome because of the inability of the patient to undergo timely source removal. This case illustrates the importance of a multidisciplinary approach of both surgical intervention and antifungal antibiotics in treating a case of FE in order to obtain a good outcome.

## Case details

A 55-year-old male, with no known co-morbidities, having a history of rheumatic heart disease with severe mitral stenosis 10 years previously, presented at our tertiary care hospital with complaints of fever and weight loss over the past 5 months. He had been admitted to a local hospital 1 month previously with a similar complaint and received ceftriaxone by injection along with symptomatic management for 10 days. He had been operated on for mitral valve regurgitation with prosthetic valve insertion 5 years previously. Since then he had been taking oral anticoagulants such as aspirin 75 mg OD, warfarin 6 mg OD, digoxin 25 mg OD (5/7) and torsemide 10 mg OD. He also had a history of undergoing right inguinal hernia repair 7 years previously. On examination, he was extremely cachexic and had grade 3 clubbing. He was febrile with an axillary temperature of 102° F and a respiratory rate of 20 min^−1^, and his blood pressure was 110/80 mm Hg in the right arm supine position. Upon examining the cardiovascular system, a precordial bulge was observed with a mid-sternal scar suggestive of previous sternotomy. On auscultation, a pansystolic murmur of grade 3/6 without ejection systolic click was heard. Per-abdominal examination revealed mild splenomegaly and left reducible indirect inguinal hernia along with a surgical scar over the same site. Other systemic examinations were normal. An urgent electrocardiogram (ECG) was ordered, which was suggestive of atrial fibrillation with a heart rate of 114 min^−1^. The chest X-ray findings were within normal limits. The patient was admitted to our hospital for further evaluation.

Upon admission, detailed physical examination was performed and the above clinical findings were confirmed. Routine blood investigations were sent, the findings of which showed haemoglobin of 9.0 gm dl^−1^, total leucocyte count of 4390 mm^−3^ with 69 % neutrophil and 28 % lymphocytes. The platelet count of 63 000 mm^−3^ and urine routine examination showed one to two pus cells per high power field. The patient’s ESR (78 mm/1st hour) and CRP (64.72 mg l^−1^) were raised. His fasting blood sugar was 94 mg dl^−1^ and HBA1c was 5.1 %. Liver function test showed mild unconjugated hyperbilirubinemia (indirect bilirubin 1.44 mg dl^−1^) with reversal of albumin (2.78 gm dl^−1^) to globulin (4.42 g dl^−1^) ratio. Kidney function tests were normal (urea 33 mg dl^−1^, creatinine 0.88 mg dl^−1^). d-dimer was 0.43 ug ml^−1^ (0–0.5 ug ml^−1^), rheumatoid factor was 13.0 U ml^−1^ (<14 U ml^−1^), PT/INR was 71.1/6.89 (11–13 s/2–3) and APTT was 67.8 (21–35 s). Serological tests for HIV, HCV and HBsAg were negative. Three sets of blood culture bottles (BACTEC, BD) and urine culture were sent and the patient was started empirically on ceftriaxone by injection (1 gm i.v. BD), vancomycin (500 mg i.v. BD) and gentamycin by injection (80 mg i.v. BD). Ultrasonography of the whole abdomen showed mild hepatomegaly (15.5 cm), splenomegaly (16 cm) with mild ascites and partially thrombosed aneurysm of ~2.8×1.9 cm of the superior mesenteric artery. CT angiography was also performed, showing normal common carotid arteries, internal carotid arteries, vertebral arteries and anterior, middle and posterior cerebral arteries of normal anatomy and calibre without any arterio-venous malformations. Fundoscopic examination was also normal. Transthoracic echo-cardiography (TTE) was performed, which showed restricted prosthetic mitral valve leaflet and vegetation over aorto-mitral continuity (dimensions: 35×9 mm) with mild aortic regurgitation (AR) and left ventricular ejection fraction (LVEF) of 55 %. Urine culture was found to be sterile after 48 h. However, all three blood cultures sent had been flagged as positive, showing growth of budding yeast cells (BYCs) on Gram stain, following which injection of liposomal amphotericin B (200 mg i.v. infusion OD) was added to the treatment regimen awaiting the identification and anti-fungal susceptibility testing (AFST) report on the BYCs. On day 4, the organism was identified as *C. parapsilosis* by matrix-assisted laser desorption/ionization time-of-flight mass spectrometry (MALDI-TOF MS) (bioMérieux, France) and it was susceptible to fluconazole (MIC=0.5 µg ml^−1^), voriconazole (MIC=0.125 µg ml^−1^), itraconazole (MIC=1 µg ml^−1^) and caspofungin (MIC=0.5 µg ml^−1^), and was intermediate for amphotericin B (MIC=4 µg ml^−1^) using the AFST E-strip method. Following this, the ongoing antimicrobial regimen was stopped and a combination of high-dose caspofungin (150 mg i.v. OD) and fluconazole (400 mg i.v. OD) was started for the patient. After 10 days of treatment, fever subsided and the patient started regaining appetite and weight. The isolate was later on reported to be susceptible to amphotericin B by broth microdilution (MIC=2 µg ml^−1^), but the patient was continued on the caspofungin and fluconazole combination because of better clinical response.

**Fig. 1.**
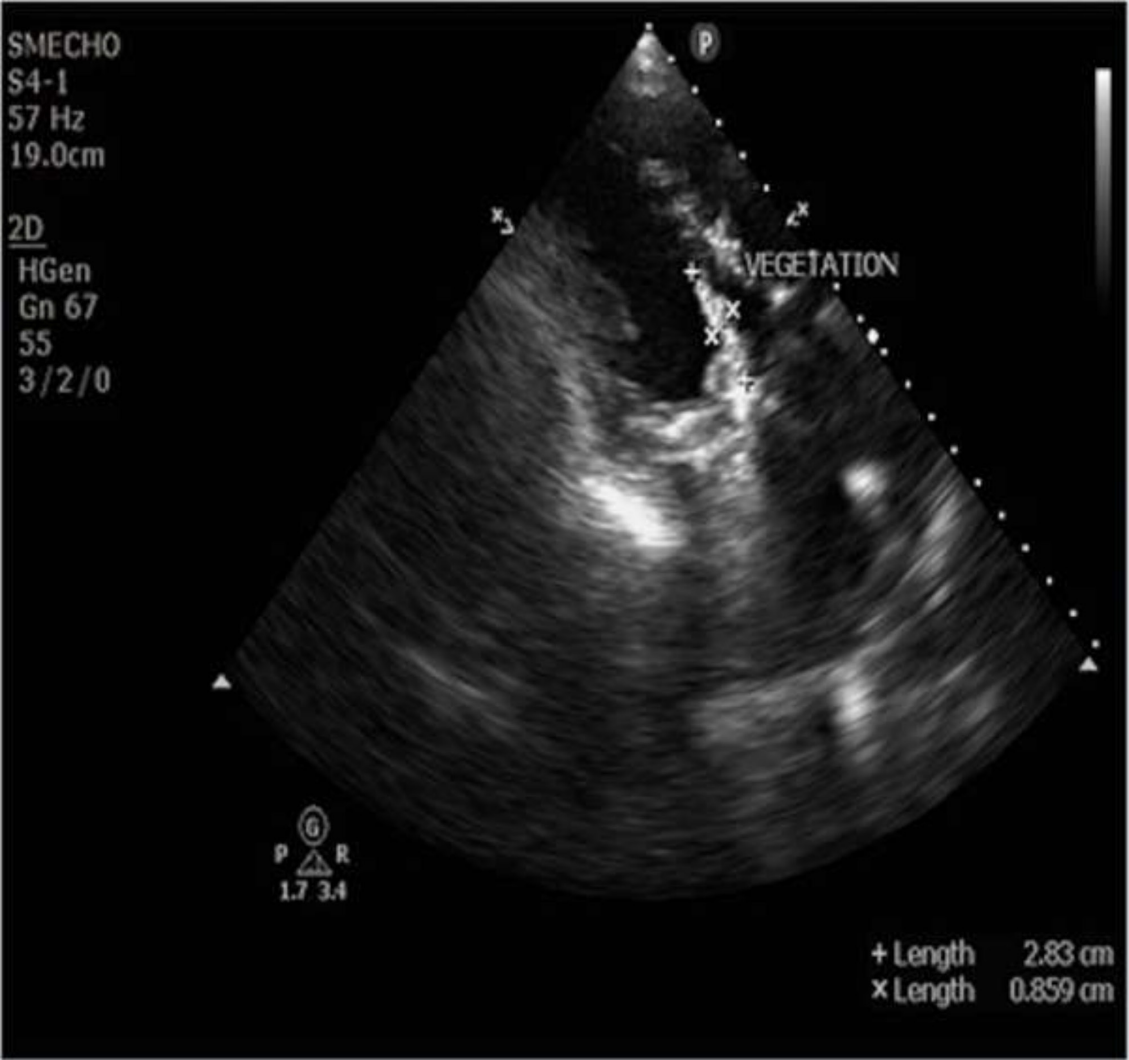
Restricted prosthetic mitral valve leaflet and vegetation over aorto-mitral continuity (28×8 mm).

On day 18 of hospitalization, the patient developed left lower limb pain and persistent headache, not relieved by analgesics. Abdominal and left lower limb arterial Doppler showed a thrombus occluding the superior mesenteric artery (2.5 cm in length) and left common femoral artery (2 cm in length extending into the superior femoral artery and dorsal femoral artery). CE-MRI brain showed acute embolic infarcts in the right fronto-parietal region, left frontal lobe and bilateral cerebellar hemispheres, along with chronic infarct in the right temporal lobe with cortical laminar necrosis in the right superior and inferior temporal gyrus and occlusion in the right middle cerebral artery in its M2 segment. Throughout the hospitalization, the patient had persistent thrombocytopenia, for which peripheral blood film examination was performed, which showed large platelets along with mild anisocytosis. Bone marrow biopsy was also performed, whose findings were otherwise normal except for some reactive changes. The bone marrow aspirate was also sent for culture in two BACTEC blood culture bottles, which again flagged positive for BYCs. However, repeat TTE performed around the same time revealed a reduced vegetation size (28×8 mm) (Fig. 1). The patient was treated with 28 days of high-dose caspofungin and fluconazole along with continuation of oral anti-coagulants. The patient was symptomatically better after at the end of 4 weeks and repeat paired blood cultures were sterile. He was discharged on fluconazole maintenance therapy (400 mg PO BD) along with oral aspirin 75 mg OD, acenocoumarol 1 mg OD, digoxin 0.25 mg (5/7) and torsemide 10 mg OD. He was advised to come back for fortnightly follow-up in order to make a prompt decision on undergoing surgical intervention. The post-discharge follow-up period was uneventful for the next two visits. After a week, the patient presented at the emergency room with respiratory distress. Cardiology reference was sought. Echocardiography was suggestive of acute heart failure and the patient succumbed to the illness.

## Discussion

*Candida* endocarditis (CE) is a rare disease that can occur following *Candida* bloodstream infections or secondary to translocation from gut flora or due to antibiotic pressure in critically ill patients [9]. A recently performed systematic review showed that *C. albicans* and *C. paraspsilosis* are the most common species causing FE [10]. Other non*-albicans Candida* species such as *C. krusei*, *C. dubliniensis*, *C. lusitaniae* and multidrug-resistant *C. auris* have also been associated with a substantial rise in in-hospital morbidity and mortality among the FE cases [5, 11].

CE commonly occurs in the setting of underlying malignancy, chronic liver disease, previous endocarditis, antimicrobial exposure, abdominal surgery or intravenous drug abuse, in the presence of a central venous catheter and also previous cardiac surgery [9]. In our case, the patient had undergone cardiac surgery and prosthetic valve replacement 5 years previously. *Candida* prosthetic valve endocarditis (PVE) is more commonly left-sided, occurs mostly at the aortic valve and is often missed by TTE because of its anatomical location [12]. Our patient had a mitral prosthetic valve vegetation and was easily caught on TTE because of its larger size [13]. It was a case of late PVE, occurring beyond 12 months of cardiac surgery [14]. Even though most of the late PVE cases are community acquired and are caused by streptococci and *Staphylococcus aureus *, followed by coagulase-negative staphylococci and enterococci [14], in our case it was due to *C. parapsilosis*. Cases of later presentation with PVE, causing candidaemia following heart valve replacement, have been reported to occur within 1 year of surgery. However, our patient had no previous history of symptoms necessitating hospitalization and possible isolation of *Candida* [12]. Hence the source of infection in the patient could not be established. Thus, it could be that an episode of candidaemia did occur but was not clinically evident before the patients presented with PVE.

The diagnosis of CE is often difficult, as most of the patients do not present with typical clinical features of IE, such as fever, splenomegaly and clubbing, and typical immunological features, such as Roth spots and Osler nodes. However, such patients are at higher risk of embolic events compared to bacterial endocarditis because of the larger and more friable vegetations [10]. Our patient was extremely cachexic and presented with fever, mild splenomegaly, grade 3 clubbing and changing murmur. Later he also developed left lower limb emboli and multiple cerebral infarcts. From the blood culture as well as the bone marrow culture of the patient, *C. parapsilosis* was isolated, which confirmed the diagnosis of CE. Usually, the diagnosis of CE is done by isolating the pathogen from blood or tissue or vegetation by culture followed by confirmation with molecular methods such as polymerase chain reaction (PCR) [10, 12]. However, culture-negative CE cases are also common, in which case serological tests such as 1,3-β-d-glucan (BDG) is a good marker of invasive candidiasis, showing 90–100 % sensitivity [15]. BDG can be used as an early diagnostic marker of CE and for prompt initiation of antifungal antibiotics after ruling out other potential causes [16].

In terms of treatment, the patient was first started on injectable liposomal amphotericin B empirically and was later shifted to a combination of high-dose caspofungin and fluconazole based on the AFST report. However, he could not be taken up for surgery immediately because he was extremely cachexic. The most recent Infectious Diseases Society of America (IDSA, 2016) [17] and American Heart Association (AHA, 2015) [18] guidelines suggest the treatment of *Candida* PVE with liposomal amphotericin B (3–5 mg/kg/day) in combination with or without flucytosine (25 mg/kg four times daily) or high-dose caspofungin (150 mg daily) as an alternative, along with valve replacement. This has to be followed by chronic suppressive therapy with fluconazole (6–12 mg/kg/day) to prevent recurrences. Echinocandins exert their fungicidal activity by inhibiting BDG synthesis and disrupting the fungal cell wall and are considered to be the drug of choice against most *Candida* species [19]. With medical therapy, the size of the vegetations was shown to reduce and the patient was afebrile. He was discharged on fluconazole maintenance therapy and it was planned for him to have surgery later. Even though lifelong fluconazole therapy has shown survival benefits in patients with *Candida* PVE who are unfit for surgery [10], the patient passed away due to cardiac failure before he could undergo valve replacement. Large vegetations (>2 cm) have been identified as an independent predictor of mortality in cases with fungal aetiology, which could have contributed to the fatality in our patient [20]. Despite the use of medical and surgical combination therapy, the mortality rate for *C. parapsilosis* endocarditis remains as high as 40 % [21]. A combined approach from the ID team and the cardiology team is recommended for timely diagnosis and proper management of FE cases in order to reduce fatality.

## Conclusion

We describe a case of *C. parapsilosis* prosthetic valve endocarditis in a patient with a history of undergoing cardiac surgery but without any other co-morbidities. The changing epidemiology of candidiasis cases indicates that invasive diseases due to this species may be much more frequent. Although the use of echinocandins and lipid formulations of amphotericin B have been promising in the management of such cases, fatality has been reported without surgical intervention. However, despite the use of a combined medical and surgical approach, the mortality due to *Candida* endocarditis still remains high. This emphasizes the need for a combined approach of a high index of clinical suspicion, early diagnosis by serological markers such as BDG followed by culture or PCR and prompt initiation of appropriate antifungals to improve the outcome.
